# Supplementary material for: The interactome of intact mitochondria by cross-linking mass spectrometry provides evidence for coexisting respiratory supercomplexes
Source: Mol Cell Proteomics. 2017 Dec 8;17(2):216–32. doi: 10.1074/mcp.RA117.000470 (PMC5795388; doi:10.1074/mcp.RA117.000470)
Supplement: Supplemental Data [file supp_RA117.000470_133922_0_supp_23978_fzffwf.zip › spectra_annotation/mito_DR_spectra_annotation/280-1-38-1-8-1.pdf]

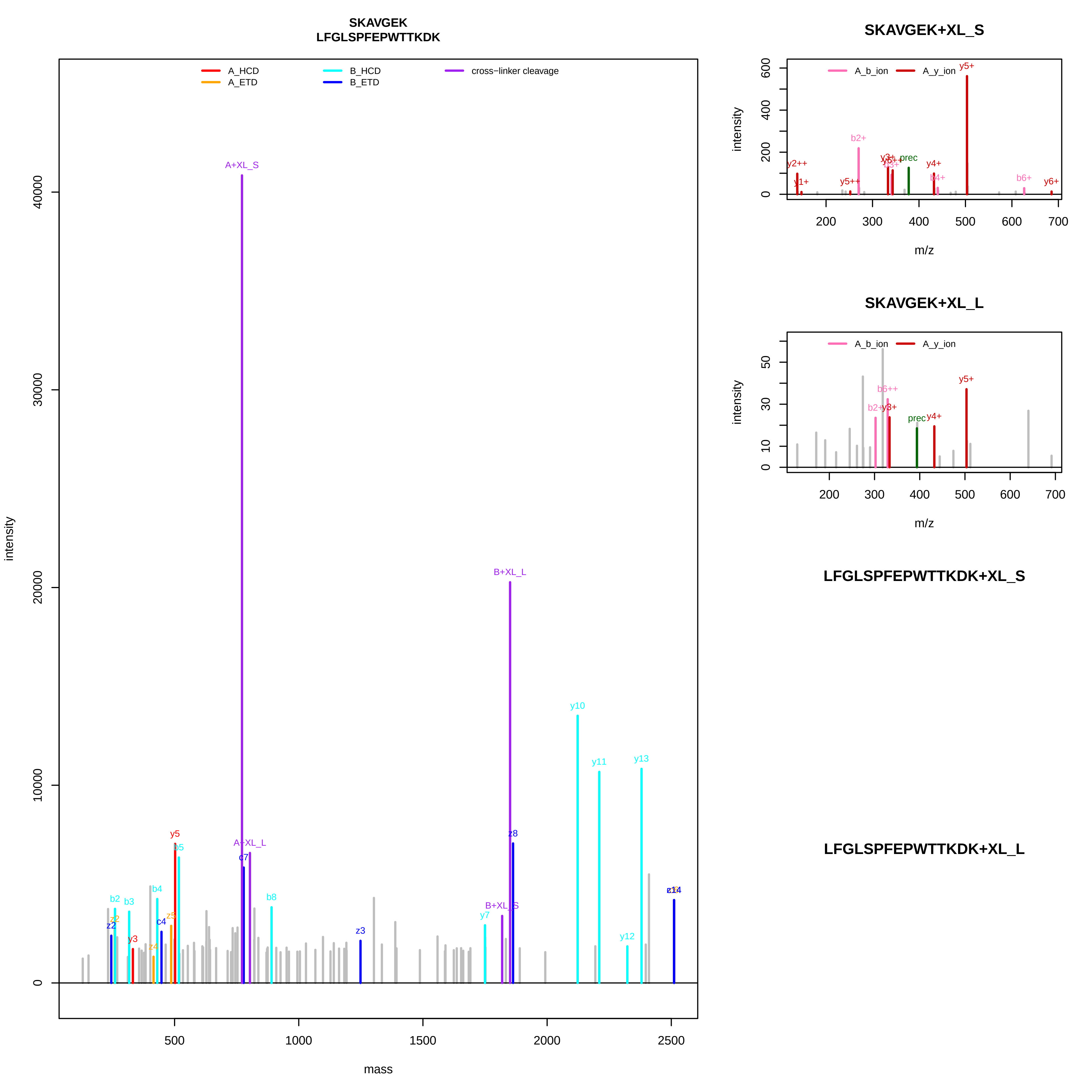
SKAVGEK
LFGLSPFEPWTTKDK
A_HCD
A_ETD
B_HCD
B_ETD
cross−linker cleavage
A+XL_S
B+XL_L
A+XL_L
B+XL_S
y10
y11
y13
y12
y7
y5
y3
b2
b3
b4
b5
b8
z2
z4
z5
c4
c7
z3
z8
z14
intensity
mass
0
10000
20000
30000
40000
500
1000
1500
2000
2500
SKAVGEK+XL_S
A_b_ion
A_y_ion
y5+
b2+
y2++
y1+
y5++
y3+
prec
y4+
b4+
b6+
y6+
m/z
SKAVGEK+XL_L
b6++
b2+
y3+
prec
y4+
y5+
LFGLSPFEPWTTKDK+XL_S
LFGLSPFEPWTTKDK+XL_L
